# The State of Perplexity During Management of Gall Bladder Malignancy in an Expectant Young Mother

**DOI:** 10.7759/cureus.13099

**Published:** 2021-02-03

**Authors:** Deepak Rajput, Amit Gupta, Sweety Gupta, Ankit Rai, Sruthi Shasheendran

**Affiliations:** 1 Department of Surgery, All India Institute of Medical Sciences Rishikesh, Rishikesh, IND; 2 Department of Radiation Oncology, All India Institute of Medical Sciences Rishikesh, Rishikesh, IND

**Keywords:** gall bladder cancer, pregnancy, preterm delivery, chemotherapy, cholecystectomy

## Abstract

During pregnancy, diagnosed cancer causes a major disturbance in the life of a pregnant woman and her family. The advanced stage of illness requiring systemic treatment inevitably leads the treating practitioner, with two lives at risk, into an ethical dilemma. The unborn child can be affected by the application of cancer medication to the mother as it is exposed to fetotoxic drugs. On the other hand, withholding therapy to allow fetal maturity may make the disease of the mother metastatic. Gall bladder carcinoma is often diagnosed as an unresectable disease (metastatic or locally advanced) due to its nonspecific symptomatology and carries the worst prognosis of any gastrointestinal or hepatobiliary neoplasm. We report a case of locally advanced gall bladder cancer (GBC) diagnosed during late pregnancy wherein the mother opted to continue the pregnancy without any intervention. A review of literature has been done to investigate the role of female hormones in a pregnancy complicating GBC with emphasis on management dilemma and the associated pitfalls.

## Introduction

Primary gall bladder cancer (GBC) is the most common malignancy of the biliary tract with a higher female predominance [1]. Pregnancy complicated by gall bladder disease is common with a prevalence between 2%-14% [2]. Elevated estrogen levels, increased cholesterol secretion, delayed gall bladder emptying, supersaturation of bile during pregnancy are the possible reasons [3]. Whether these factors can complicate the malignancy of the biliary tract or initiate the process of carcinogenesis during pregnancy is unclear.

There are few reported cases of resectable gall bladder malignancies complicating a pregnancy. Mendoza et al. reported a case of incidentally diagnosed in-situ GBC during pregnancy for which the patient underwent open cholecystectomy at 33 weeks of gestation [3]. Attraplsi et al. reported another case of GBC in a young female with Crohn’s disease during pregnancy. The patient underwent laparoscopic cholecystectomy, was delivered and started on adjuvant chemotherapy [4]. We highlight the management dilemma associated with GBC diagnosed during pregnancy.

## Case presentation

A 26-year-old primigravida, with an unremarkable 28 weeks gestation, presented to our institute with dull aching pain over the right upper abdomen and associated nausea and loss of appetite for the last one month. The patient also had gone to a private hospital three months prior, with a ten-day duration of pain in the abdomen and jaundice, where she underwent an endoscopic retrograde cholangiopancreatography (ERCP) and bile duct stone retrieval followed by a stent placement for suspected choledocholithiasis. She had no post-procedural complications. She had a body mass index (BMI) of 20.4, was a non-smoker, and gave no family history of biliary tract malignancy. She gave no history of gallstone disease before the pregnancy. Physical examination revealed non-tender hepatomegaly with gravid uterus corresponding to 24 weeks gestation. On inquiry from the husband, her previous antenatal ultrasounds were normal for fetal growth.

**Figure 1:**
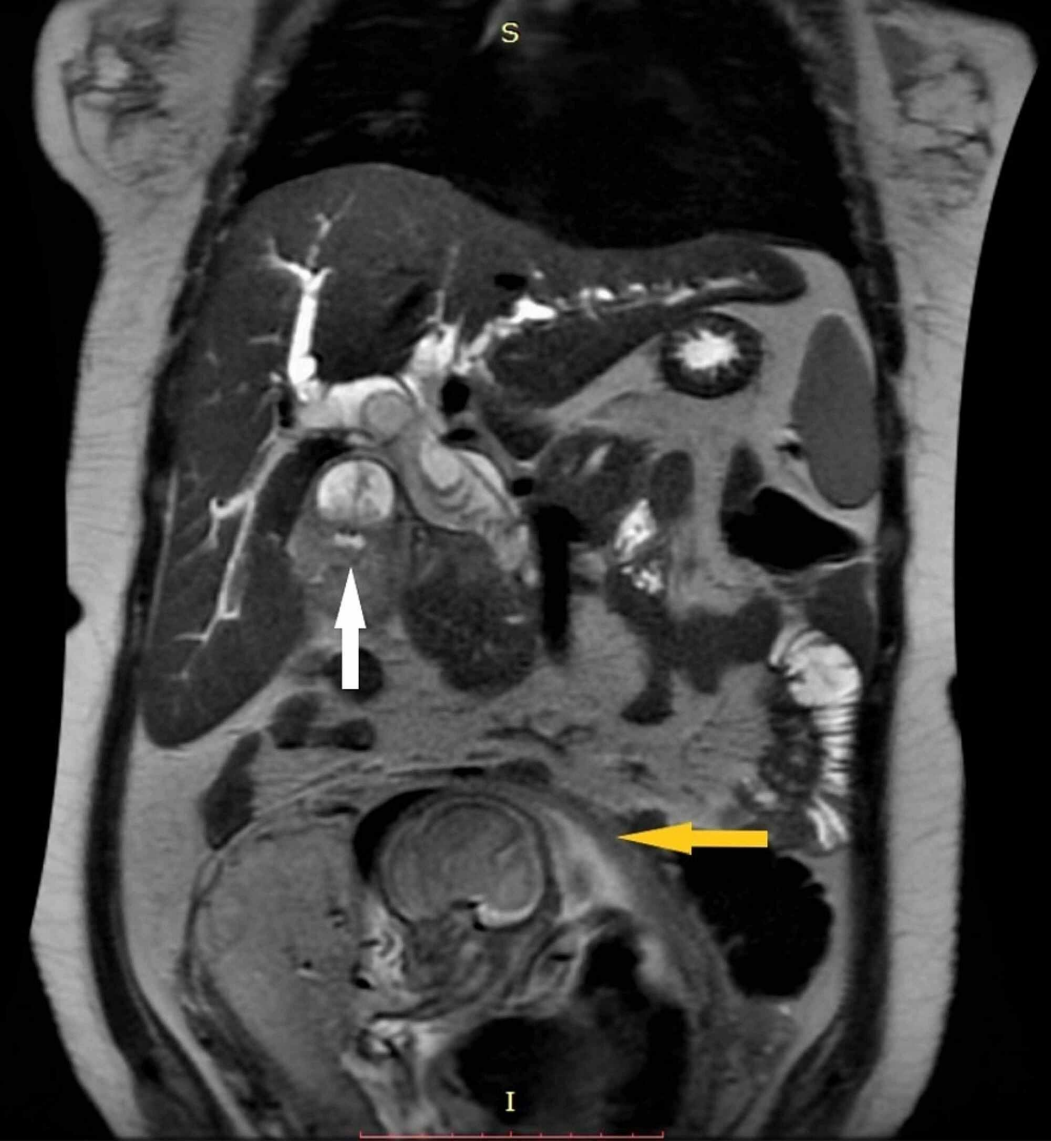
MRCP showing polypoidal gall bladder mass (white arrow), CBD dilatation with a stent in situ, and gravid uterus (yellow arrow) MRCP: magnetic resonance cholangiopancreatography; CBD: common bile duct.

Initial laboratory results were as follows: Hb 8.9 g/dL, white blood cell (WBC) count 4.7 x 10^9^/L, total bilirubin 62.7 µmol/L, direct bilirubin 44 µmol/L, alanine aminotransferase (ALT) 152 U/L, aspartate aminotransferase (AST) 138 U/L, and alkaline phosphatase (ALP) 1098 U/L. The hepatitis panel was negative. Serum tumor markers (cancer antigen (CA) 19-9, carcinoembryonic antigen (CEA), CA 125) were also sent; of which only CA 125 showed slight elevation (69.05 U/mL). An endoscopic ultrasound (EUS) done, to evaluate the lower end of the bile duct, revealed a 4.4 x 2.9 cm sized polypoidal lesion at the gall bladder neck that showed dysplastic cells on cytological examination. The patient was further evaluated by a magnetic resonance cholangiopancreatography (MRCP), which revealed locally advanced GBC and a bile duct stent in situ (Figure 1).

The patient and her husband were counseled about the aggressive nature of the illness and its prognosis and offered multidisciplinary care. She was advised about starting chemotherapy as the disease might turn metastatic in due course of time, and the risks to the fetus against the benefits of the mother’s treatment were discussed. The patient continued with her pregnancy and was lost to follow-up. Confounding factors in her decision making were cancer stage and prognosis, first child, potential teratogenic effects of chemotherapy, and socio-cultural reasons. She had a cesarean section and delivered a late preterm infant at 34 weeks gestation at an outside nearby facility after prophylactic antenatal steroid administration. The birth weight of the neonate was 2.2 kg and the child was kept in a neonatal intensive care unit (NICU) for 24 hours. The mother could breastfeed her child on a subsequent day. The baby developed physiological jaundice on the third day, which showed a spontaneous decline after the fifth day.

**Figure 2:**
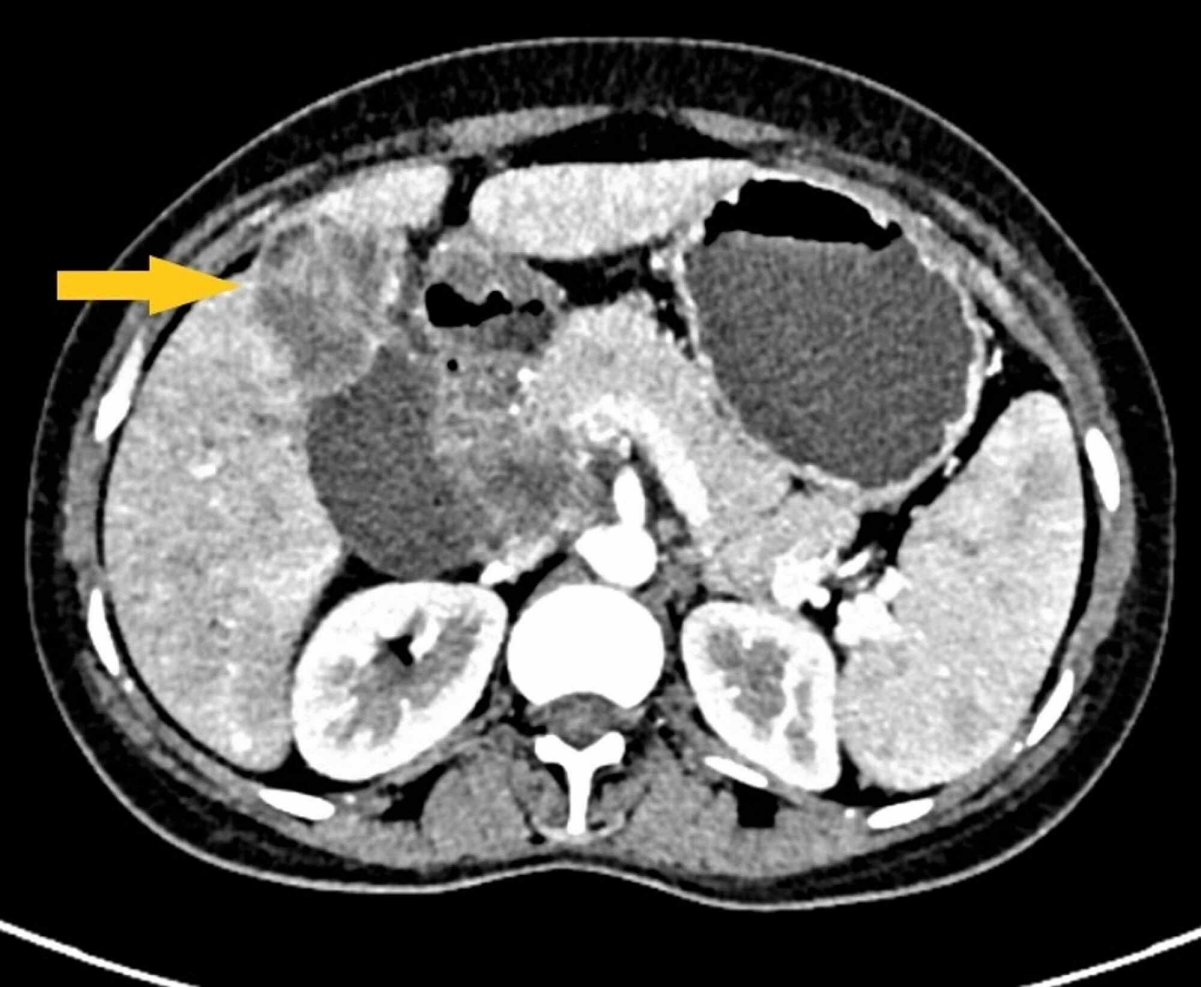
CECT abdomen showing an exophytic gallbladder mass infiltrating liver parenchyma (yellow arrow) CECT: contrast-enhanced computed tomography.

Two weeks post-delivery, the patient came back to our emergency with severe acute cholangitis. An ultrasound report, done in the 34th week to look for fetal well being, showed multiple space-occupying lesions in the liver. Hence a contrast-enhanced computed tomography (CECT) scan of the abdomen and thorax was done that revealed advanced GBC with metastatic deposits in the liver (Figure 2).

An urgent biliary decompression was achieved via a percutaneous trans-hepatic biliary drainage (PTBD) catheter placed under radiological control. Image-guided biopsy from the gall bladder mass showed adenocarcinoma and the patient was planned for chemotherapy. However, the patient continued to deteriorate with worsening biliary sepsis and finally succumbed to her illness over the next 12 days.

## Discussion

GBC is the most aggressive of all biliary tract cancers with the shortest median survival of six months and a five-year survival rate of only 5%, with resection as the only curative treatment [5]. The apparent association of multiple full-term pregnancies and the protective effect of early menopause suggest a putative role of female hormones in the pathogenesis of biliary tract cancers [6].

Lambe et al. studied the effect of parity on biliary tract cancers and found a positive relationship between the number of live births and GBC. However, this association becomes weaker and non-significant if age at first birth is also included. The authors thus concluded that parity increases the risk of cancer only if first birth occurs below 25 years of age [7]. This can be explained by the observations of Panagiotopoulou et al. that pregnancy estrogens are highest when the first pregnancy occurs between the age group of 20-24 years and decreases progressively with increasing age at first birth [8]. Also, early first pregnancy may allow gallstone induced carcinogenesis to proceed till completion. Similar findings were reported by Vecchia et al., with 1.9 times increased risk of GBC in multipara [9].

Moerman et al., in a case-control study, concluded that early menarche and first pregnancy, higher parity, and prolonged fertility were associated with increased risk of biliary tract cancers [10]. However, in another study, Andreotti et al. observed that late age at menarche is associated with GBC [11].

GBC may present as focal or diffuse asymmetric wall thickening in 30%-40% of cases [12]. However, differentiating between the various causes of gall bladder wall thickening such as acute and chronic cholecystitis, GBC, and other nonspecific causes such as ascites, congestive heart failure, and hypoalbuminemia may be difficult.

Ultrasound abdomen is the primary imaging modality for evaluating gall bladder diseases, however, it fails to distinguish between benign and malignant gall bladder wall thickening accurately [13]. Dual-phase helical CT can determine resectability in GBC with an accuracy of up to 93% whereas, MRCP with three-dimensional magnetic resonance (MR) angiography quite accurately detects vascular invasion, with sensitivity and specificity approaching 100% and 87%, respectively [14]. MR without contrast is the radiological imaging of choice in pregnancy. Mitake et al. reported the effectiveness of endoscopic ultrasonography (EUS) in the diagnosis of GBC and the determination of the extent of tumor invasion [15]. Elastography combined with sonography can be used as the primary imaging tool for diagnosing early GBC, especially in pregnancy [16].

GBC is characterized by local and vascular invasion, extensive regional lymph nodes, and distant metastases [17]. The standard treatment for unresectable disease is fluoropyrimidine-based or gemcitabine-based chemotherapy. Wiesweg et al. reported the use of gemcitabine and cisplatin in the 18th week of pregnancy in a 38-year-old woman diagnosed with metastatic adenocarcinoma of the biliary tract [18]. Those with locally advanced GBC may respond to newer adjuvant combination chemotherapy and molecular targeted therapeutic options. National Comprehensive Cancer Network (NCCN) now recommends microsatellite instability (MSI) and/or mismatch repair (MMR) testing in patients with unresectable or metastatic GBC as those with high microsatellite instability (MSI-H) or MMR deficiency (dMMR) may benefit from targeted therapy with pembrolizumab [19].

After organogenesis during the first trimester, the eyes, teeth, ears, palate, genitalia, hematopoietic system, and central nervous system continue to mature in the fetus until term. Risks to the fetus during the second and third trimesters are intrauterine growth limits, low birth weight, premature delivery, functional abnormalities, and adverse effects of antineoplastics on the mother, such as myelosuppression. The FOLFOX regime (5-fluorouracil, oxaliplatin, and leucovorin), used as second-line chemotherapy in advanced/metastatic biliary tract cancers (ABC-06 trial), has demonstrated a low percentage or minimal congenital malformations when administered during the second and third trimester in pregnant patients with colorectal cancer [20]. Multidisciplinary care with physical and emotional support is crucial for the management of the biliary tract and most other malignancies in pregnancy.

## Conclusions

GBC is an aggressive malignancy with a poor prognosis. MR without contrast is the radiological imaging of choice in pregnancy. Pregnancy complicated with GBC limits the treatment option. A resectable GBC during pregnancy should be treated by surgery, preferably in the second trimester after appropriate optimization. The unresectable disease presents a management dilemma as most patients prefer to continue the pregnancy, which also causes the disease to progress. Chemotherapy though not recommended during the first trimester, may be administered in the second and third trimesters with limited fetal risk but not three weeks before expected delivery.

## References

[1] Carriaga MT, Henson DE (1995). Liver, gallbladder, extrahepatic bile ducts, and pancreas. Cancer.

[2] Schwulst SJ, Son M (2020). Management of gallstone disease during pregnancy. JAMA Surg.

[3] Mendoza GO, Sandoval JM, Orozco VMM, Hernández M (2005). Complicated pregnancy with lithiasis and resectable gallbladder cancer. A case report and literature review. Ginecol Obstet Mex.

[4] Attraplsi S, Shobar RM, Lamzabi I, Abraham R (2013). Gallbladder carcinoma in a pregnant patient with Crohn's disease complicated with gallbladder involvement. World J Gastrointest Oncol.

[5] Miller G, Jarnagin WR (2008). Gallbladder carcinoma. Eur J Surg Oncol.

[6] Tavani A, Negri E, La Vecchia C (1996). Menstrual and reproductive factors and biliary tract cancers. Eur J Cancer Prev.

[7] Lambe M, Trichopoulos D, Hsieh CC, Ekbom A, Adami HO, Pavia M (1993). Parity and cancers of the gallbladder and the extrahepatic bile ducts. Int J Cancer.

[8] Panagiotopoulou K, Katsouyanni K, Petridou E, Garas Y, Tzonou A, Trichopoulos D (1990). Maternal age, parity, and pregnancy estrogens. Cancer Causes Control.

[9] La Vecchia C, Negri E, Franceschi S, Parazzini F (1993). Long-term impact of reproductive factors on cancer risk. Int J Cancer.

[10] Moerman CJ, Berns MP, Bueno de Messqita HB, Runia A (1994). Reproductive history and cancer of the biliary tract in women. Int J Cancer.

[11] Andreotti G, Hou L, Gao YT (2010). Reproductive factors and risks of biliary tract cancers and stones: a population-based study in Shanghai, China. Br J Cancer.

[12] Sons HU, Borchard F, Joel BS (1985). Carcinoma of the gallbladder: autopsy findings in 287 cases and review of the literature. J Surg Oncol.

[13] Shlaer WJ, Leopold GR, Scheible FW (1981). Sonography of the thickened gallbladder wall: a nonspecific finding. AJR Am J Roentgenol.

[14] Reid KM, Ramos-De la Medina A, Donohue JH (2007). Diagnosis and surgical management of gallbladder cancer: a review. J Gastrointest Surg.

[15] Mitake M, Nakazawa S, Naitoh Y (1990). Endoscopic ultrasonography in diagnosis of the extent of gallbladder carcinoma. Gastrointest Endosc.

[16] Kapoor A, Kapoor A, Mahajan G (2011). Differentiating malignant from benign thickening of the gallbladder wall by the use of acoustic radiation force impulse elastography. J Ultras Med.

[17] Jarnagin WR, Ruo L, Little SA (2008). Patterns of initial disease recurrence after resection of gallbladder carcinoma and hilar cholangiocarcinoma: implications for adjuvant therapeutic strategies. Cancer.

[18] Wiesweg M, Aydin S, Koeninger A (2014). Administration of gemcitabine for metastatic adenocarcinoma during pregnancy: a case report and review of the literature. AJP Rep.

[19] Le DT, Durham JN, Smith KN (2017). Mismatch repair deficiency predicts response of solid tumors to PD-1 blockade. Science.

[20] Rogers JE, Dasari A, Eng C (2016). The treatment of colorectal cancer during pregnancy: cytotoxic chemotherapy and targeted therapy challenges. Oncologist.

